# Baseline Prevalence of Trachoma in Refugee Settlements in Uganda: Results of 11 Population-based Surveys

**DOI:** 10.1080/09286586.2021.1961816

**Published:** 2021-09-06

**Authors:** Gilbert Baayenda, Francis Mugume, Alfred Mubangizi, Patrick Turyaguma, Edridah M. Tukahebwa, Sarah Byakika, Binta Kahwa, Darlson Kusasira, Ana Bakhtiari, Sarah Boyd, Robert Butcher, Anthony W. Solomon, Ben Binagwa, Stella Agunyo, Martin Osilo, Kathryn Crowley, Wangeci Thuo, Mike French, Elizabeth Plunkett, Aryc W. Mosher, Emma M. Harding-Esch, Jeremiah Ngondi

**Affiliations:** a Ministry of Health, Kampala, Uganda; b Kampala International University Medical School, Kampala, Uganda; c Refugees Department, Office of the Prime Minister, Kampala, Uganda; d Task Force for Global Health, Atlanta, Georgia, USA; e Clinical Research Department, London School of Hygiene & Tropical Medicine, London, UK; f Department of Control of Neglected Tropical Diseases, World Health Organization, Geneva, Switzerland; g RTI International, Kampala, Uganda; h RTI International, Washington DC, USA; i United States Agency for International Development, Washington DC, USA

**Keywords:** Trachoma, prevalence, refugee, Uganda, neglected tropical diseases

## Abstract

**Purpose:**

There are several settlements in the Northern and Western Regions of Uganda serving refugees from South Sudan and Democratic Republic of Congo (DRC), respectively. Trachoma prevalence surveys were conducted in a number of those settlements with the aim of determining whether interventions for trachoma are required.

**Methods:**

An evaluation unit (EU) was defined as all refugee settlements in one district. Cross-sectional population-based trachoma prevalence survey methodologies designed to adhere to World Health Organization recommendations were deployed in 11 EUs to assess prevalence of trachomatous inflammation—follicular (TF) in 1–9-year-olds and trachomatous trichiasis (TT) unknown to the health system in ≥15-year-olds. Household-level water, sanitation and hygiene coverage was also assessed in study populations.

**Results:**

A total of 40,892 people were examined across 11 EUs between 2018 and 2020. The prevalence of TF in 1–9-year-olds was <5% in all EUs surveyed. The prevalence of trachomatous trichiasis (TT) unknown to the health system in ≥15-year-olds was <0.2% in 5 out of 11 EUs surveyed and ≥0.2% in the remaining 6 EUs. A high proportion of households had improved water sources, but a low proportion had improved latrines or quickly (within a 30-minute return journey) accessible water sources.

**Conclusions:**

Implementation of the antibiotic, facial cleanliness and environmental improvement components of the SAFE strategy is not needed for the purposes of trachoma’s elimination as a public health problem in these refugee settlements; however, intervention with TT surgery is needed in six EUs. Since instability continues to drive displacement of people from South Sudan and DRC into Uganda, there is likely to be a high rate of new arrivals to the settlements over the coming years. These populations may therefore have trachoma surveillance needs that are distinct from the surrounding non-refugee communities.

## Introduction

Trachoma is the world’s leading infectious cause of blindness.^1^ It can be effectively eliminated as a public health problem with the World Health Organization (WHO)-recommended SAFE strategy,^2,3^ which includes: Surgery for trichiasis; Antibiotics to treat *Chlamydia trachomatis* infection; and Facial cleanliness and Environmental improvement to reduce *C. trachomatis* transmission.^4^ WHO has targeted trachoma for elimination as a public health problem globally within this decade in the Neglected Tropical Diseases Road Map 2021–2030.^5^ To achieve this, the prevalence of trachomatous inflammation—follicular (TF) in children aged 1–9 years must be <5% in all previously endemic districts at least two years after cessation of antibiotic mass drug administration (MDA), the prevalence of trachomatous trichiasis (TT) unknown to the health system must be less than 0.2% in individuals aged ≥15 years, and there must be a strategy in place to identify and manage incident cases of TT.^6^ Since 2012, there has been a dramatic scale-up of global efforts to determine prevalence and initiate interventions in all endemic districts in a concerted attempt to reach elimination targets.^7^ Validation of elimination of trachoma as a public health problem in several countries and the massive reduction in the global population at risk of trachoma are evidence of the positive impact of these efforts.^8^

Uganda has a well-established trachoma elimination programme and has made substantial progress towards trachoma elimination through implementation of the SAFE strategy. Baseline mapping of trachoma in suspected-endemic districts took place between 2006–2014. The TF prevalence in 1–9-year-olds was ≥5% in 51 districts and was 66% in the highest-prevalence district. As the results of the 2006–2014 surveys emerged, the Uganda Ministry of Health started azithromycin MDA in line with WHO guidance; the first treatment rounds were delivered in 2007. As interventions progressed, the Ministry and partners co-developed a plan to progress the trachoma elimination agenda in un-surveyed areas. A number of initiatives were started, including trachoma rapid assessment (TRA^9,10^) in areas adjacent to formerly endemic districts. Because of the high prevalence of trachoma in some parts of South Sudan,^11,12^ all refugee camps hosting refugees from South Sudan were invited to be assessed through TRA. Between November 2017 and February 2018, TRAs were conducted in 29 refugee settlements hosting South Sudanese refugees to determine whether population-based prevalence surveys (PBPS) were warranted. PBPS were deemed necessary where the proportion of children with TF was more than 5% in any refugee settlement in which a TRA was conducted. There were also many refugee settlements housing refugees from the Democratic Republic of the Congo (DRC) in the Western Region of Uganda but very few data from the resident Ugandan population as the region was not suspected to be endemic; because trachoma was known to be endemic in districts over the border in DRC,^13^ these settlements were also recommended for PBPS.

The objective of these baseline surveys was to estimate the prevalence of TF in children aged 1–9 years, the prevalence of TT in people aged ≥15 years and coverage of water, sanitation and hygiene (WASH) infrastructure in suspected-trachoma-endemic refugee settlements in Uganda serving refugees from South Sudan and DRC.

## Methods

### Study design

The refugee population of identified districts ranged from 5,435–287,801 people. Even though districts at either end of this range had refugee populations lesser than or greater than, respectively, the template 100,000–250,000 population for evaluation units (EUs), to align with WHO recommendations for implementation units for the SAFE strategy,^14^ an EU was defined here as all refugee settlements within a given district. In some cases, that involved a single settlement. In other cases, it involved multiple settlements.

The surveys presented in this publication were carried out in two separate series. The first took place in 2018; the second in 2020 (Table 1). Due to updates to recommendations for trachoma prevalence surveys between the two series,^15,16^ different design effects for TF^17^ were used for the two time points.

**Table 1.** Individuals aged ≥1 year enumerated and examined in refugee settlements in Uganda during baseline trachoma surveys, April 2018–February 2020.

Region	District hosting refugee settlements	Refugee settlements	EU ID	Date completed	Enumerated	Refused	Absent	Examined	Examined female (%)
Northern	Arua	Alizi, Ariwa, Lia, Ocea, Odupu, Ofuwa, Siripi, Tika	10617	April 2018	4,423	1	289	4,133	2,257 (55)
Northern	Yumbe	Bidi Bidi Zone 4; Kululu, Zone 3, Bidibidi Zone 1, Bidibidi Zone 2, Bidibidi Zone 5	10618	April 2018	4,305	2	198	4,105	2,292 (56)
Northern	Moyo	Ibakwe; Itula Zone 1; Morobi, Palorinya	10619	April 2018	3,976	2	244	3,730	2,164 (58)
Northern	Adjumani	Nyumanzi, Pagirinya, Agojo, Ailo1, Ailo 2, Alele, Baratuku, Maji 1, Maji 2 Maji 3, Mungula1 Mungula 2, mirieyi, leleme	10620	April 2018	3,703	0	206	3,497	2,109 (60)
Northern	Lamwo	Palabek	10621	May 2018	3,620	0	123	3,497	1,932 (55)
Western	Kiryandongo	Kiryandongo	10622	May 2018	3,995	0	207	3,788	2,133 (56)
Northern	Koboko	Lobule	10828	February 2020	2,354	1	200	2,153	1,175 (55)
Western	Isingiro	Nyakivaale & Oruchinga	10829	February 2020	4,248	2	108	4,138	2,217 (54)
Western	Kamwengye	Rwamwanja	10830	February 2020	4,303	2	112	4,189	2,288 (55)
Western	Kikuube	Kyangwali	10831	February 2020	3,831	5	184	3,642	2,119 (58)
Western	Kyegegwa	Kyaka II	10832	February 2020	4215	6	189	4,020	2,234 (56)
*Total*	-		-	-	*42,973*	*21*	*2060*	*40,892*	*22,920 (56)*

EU: Evaluation unit.

Both 2018 and 2020 survey series aimed to detect an anticipated TF prevalence in 1–9-year-olds of 10% with an absolute precision of ± 3% at the 95% confidence level in each EU. In all surveys, an inflation factor of 1.2 to account for non-response was applied. In both survey series, the primary sampling unit (PSU) was a settlement block, as defined by UNHCR,^18^ and the secondary sampling unit (SSU) was the household.

In 2018, there were five EUs in the Northern Region and one EU in the Western Region. In the planning for the 2018 surveys, a design effect of 2.65 was used. The calculated minimum sample size was 1,222 children aged 1–9 years per EU in 2018 EUs. Survey teams in 2018 EUs were expected to be able to recruit 35 households a day. Based on the mean expected number of children per household across all settlements, 24 PSUs per EU were visited in the 2018 survey series.

In the 2020 surveys, there was one EU in the Northern Region and four EUs in the Western Region. A design effect of 3.69 was applied to the target sample size when planning the surveys. Survey teams in these EUs were expected to be able to recruit 30 households a day. The calculated minimum sample size was 1,701 children aged 1–9 years per EU in three of the 2020 EUs (Nyakivaale & Oruchinga, Kyangwali and Kyaka II). This meant a total of 30 PSUs per EU were required in Nyakivaale & Oruchinga, Kyangwali and Kyaka II to reach the required sample size. There were two districts in the 2020 survey series with small populations (<100,000) and a finite population correction^19^ was applied there to appropriately adjust the sample size. In the refugee settlements in Rwamwanja, 1,620 children were targeted, for which 30 PSUs were thought to be sufficient. In the refugee settlements in Lobule, 1,030 children were targeted, for which 18 PSUs were selected.

In all EUs, PSUs were selected from a full list of blocks in each settlement, provided by UNHCR/OPM. PSUs for each settlement were systematically selected with probability of selection proportional to their population size. Where multiple settlements needed to be surveyed in a single EU, the number of PSUs from each settlement was proportional to the population of each settlement. Households (defined as people living together and sharing meals) were selected using a compact segment sampling method. All consenting individuals aged ≥1 year who had lived in the respective settlements for at least 1 year were eligible for enrolment. Where household members aged 1–9 years were absent at the time of the team’s first visit to the household, one return visit was made at the end of the day to increase chances of enrolment.

Standard quality assurance and quality control measures^20^ were implemented throughout each survey.

### Study ethics

Relevant authorities were consulted before surveys were initiated, including the United Nations High Commissioner for Refugees (UNHCR), the Office of the Prime Minister (OPM) and the administrative authorities of districts hosting the refugees. The Vector Control Division Research and Ethics Committee deemed the surveys to be programmatic work and therefore granted a waiver of approval. Ethical approval for Tropical Data to provide support for these surveys was granted by the London School of Hygiene & Tropical Medicine (16105). Permission for households to be enrolled in the surveys was sought from heads of each selected household. Informed verbal consent for examination was sought from each survey participant aged ≥18 years or, in the case of people aged <18 years, their parent or guardian. Additionally, children aged 6–17 years were asked to assent to take part. Consent for examination was recorded in the Tropical Data app used for data collection. Individuals found to have TF were treated with two tubes of 1% tetracycline eye ointment and instructed to apply twice daily for six weeks, provided at no cost to recipients. Participants with TT were referred to the nearest health facility for appropriate management, also provided free of charge to the participants.

### Clinical and demographic data collection

Fieldwork took place in April 2018–February 2020. The SAFE strategy had not been implemented in these settlements before these surveys took place. Before taking part in the survey, graders and recorders were required to pass internationally standardised training using the Tropical Data system.^21,22^ Data were collected and recorded in the field by certified graders and recorders.

All consenting participants ≥1 year were examined for clinical signs of trachoma using 2.5× binocular loupes. Participants were graded for TT, TF and TI using the WHO simplified grading system^23,24^ and in 2020 EUs, follicle size guides^25^ fixed to graders’ thumbnails to help maintain standardized diagnosis of TF. In the 2018 series of surveys (Table 1), distinction between the eyelid (upper or lower) from which any deviated eyelashes originated was not recorded. In response to recommendations on the definition of TT made during the fourth Global Scientific Meeting on trachoma and subsequent amendments to the WHO simplified grading scheme,^24,26^ the distinction between upper and lower eyelid trichiasis was recorded in surveys which took place in 2020 (Table 1). Regardless of from which eyelid any in-turned eyelashes arose, participants from both series who were identified with trichiasis were additionally assessed for trachomatous scarring (TS)^23^ of the upper tarsal conjunctiva, were asked questions about whether they had previously been offered management for their trichiasis and whether they had accepted surgery or epilation. TT was defined according to contemporary guidelines at the time of each survey. In the 2018 survey series, TT was called when at least one eyelash (eyelid not specified) was touching the eyeball or there was evidence of recent removal of in-turned lashes. In the 2020 series, TT was called when at least one eyelash from the upper eyelid was touching the eyeball, or there was evidence of recent removal of in-turned upper eyelid eyelashes. TS status was not considered for either definition of TT. Examiners cleaned their hands with hand sanitiser in between participants to prevent carry-over of pathogens.

### Water access, sanitation and hygiene coverage

Global positioning satellite coordinates were recorded at each surveyed household. A questionnaire on the proximity and type of WASH facilities was administered to household heads or their nominees. The questionnaire was an amended version of the WHO/ United Nations Children’s Fund (UNICEF) Joint Monitoring Programme (JMP) household questionnaire adapted for trachoma surveys.^15,21,22^ Due to updates to the Tropical Data WASH questionnaire made between 2018 and 2020, questions on handwash facilities differed between the 2018 and 2020 survey series. The findings of the survey were then summarised using WHO/UNICEF JMP definitions of water sources and sanitation facilities.^27^

In the 2018 survey series, the questionnaire required data recorders to observe whether there was a handwash facility within 10metres of a latrine. Where there was no latrine, the question was not asked. In the 2020 survey series, recorders were asked to observe whether there was a handwash facility on or near the household premises. In both questionnaires, identified handwash facilities were checked for availability of soap and water.

### Data analysis

Unadjusted prevalence estimates, adjusted prevalence estimates and confidence intervals of TF and TT unknown to the health system in key indicator age groups (1–9-year-olds and ≥15-years-olds, respectively) were generated using analysis protocols standardised for the Global Trachoma Mapping Project and Tropical Data.^15^ Association between TF (dependent variable) and age, gender and household-level variables (independent variables) was assessed using a mixed-effects binomial regression model as described elsewhere^28,29^ with EU and PSU of residence included as random-effect variables. The association between TT, age and gender was tested using a mixed-effects model with PSU of residence included as a random effect variable. For the association between TT, age and gender, TT was defined as upper and/or lower lid TT to ensure data from all EUs could be included. Model comparisons were carried out using likelihood ratio tests, and *p*-values <0.05 were considered significant.

## Results

### Study population

There were 42,973 people aged ≥1 year enumerated in 11 EUs. This included 18,480 participants aged ≥15 years and 20,589 participants aged 1–9 years. 40,892 (95%) of the enumerated individuals were examined. The response rate was 98% and 93% among 1–9-year-olds and ≥15-year-olds, respectively. The numbers of participants enumerated and examined in each EU are shown in Table 1.

### Clinical signs of disease

A total of 336 children aged 1–9 years were identified with TF, and 72 were identified with trachomatous inflammation—intense (TI) across all 11 EUs. The adjusted EU-level prevalence of TF in 1–9-year-olds was <5% in all EUs and ranged from 0.8–3.6%. The number of children with active (inflammatory) trachoma and the adjusted prevalence of disease in each EU is shown in Table 2. Among children aged 1–9 years, TF was less common in those aged 7–9 years than younger children (odds ratio [OR]: 0.58, 95% confidence interval [CI] 0.44–0.77). TF was not associated with any other individual- or household-level variables (Supplementary Table 1).

**Table 2.** Children aged 1–9 years with active (inflammatory) trachoma in refugee settlements in Uganda, April 2018–February 2020.

Region	District hosting refugee settlements	Number of 1–9-year-olds examined	Number of 1–9-year-olds with TF	Number of 1–9-year-olds with TI	Adjusted TF prevalence (%, 95% CI)
Northern	Arua	2,177	32	6	1.44 (0.94─2.13)
Northern	Yumbe	2,055	37	5	1.79 (0.84─2.93)
Northern	Moyo	1,904	14	4	0.79 (0.32─1.39)
Northern	Adjumani	1,865	33	2	1.95 (0.81─3.55)
Northern	Lamwo	1,821	30	3	1.80 (0.77─3.13)
Western	Kiryandongo	1,905	23	4	1.24 (0.66─1.78)
Northern	Koboko	909	14	8	1.29 (0.25─3.01)
Western	Isingiro	1,935	72	18	3.56 (1.89─5.40)
Western	Kamwengye	1,978	16	1	0.82 (0.36─1.43)
Western	Kikuube	1,676	37	13	1.97 (0.92─2.91)
Western	Kyegegwa	1,904	28	8	1.29 (0.74─2.01)
*Total*		*20,129*	*336*	*72*	-

**Figure 1.**
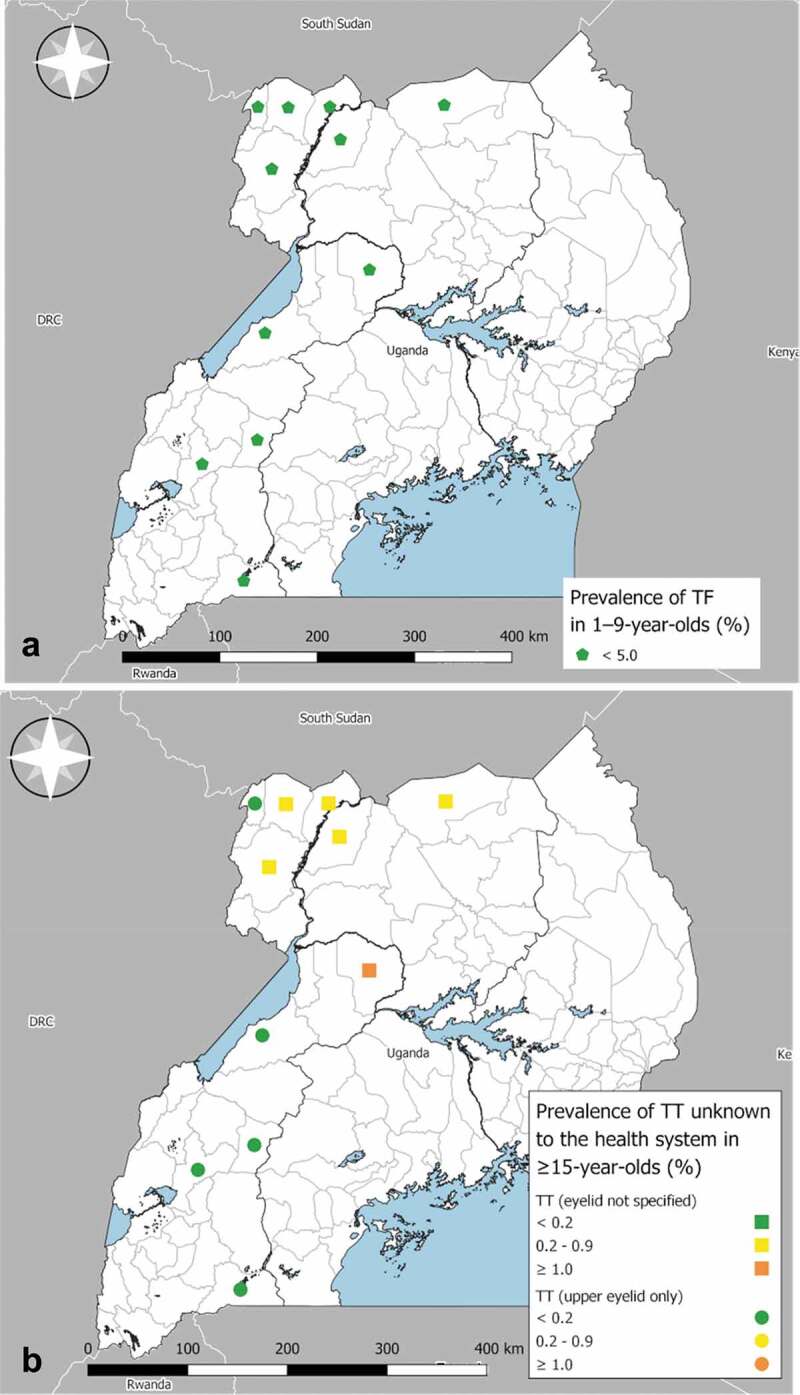
Prevalence of (a) trachomatous inflammation—follicular (TF) in 1–9-year-olds, and (b) trachomatous trichiasis (TT) unknown to the health system in ≥15-year-olds, in refugee settlements in Uganda, April 2018–February 2020. The definition of an evaluation unit in these surveys was all refugee settlements within a given district. Coloured symbols are positioned at the centroid of districts from which refugee settlements were sampled. The boundaries and names shown and the designations used on these maps do not imply the expression of any opinion whatsoever on the part of the authors, or the institutions with which they are affiliated, concerning the legal status of any country, territory, city or area or of its authorities, or concerning the delimitation of its frontiers or boundaries.

**Figure 2.**
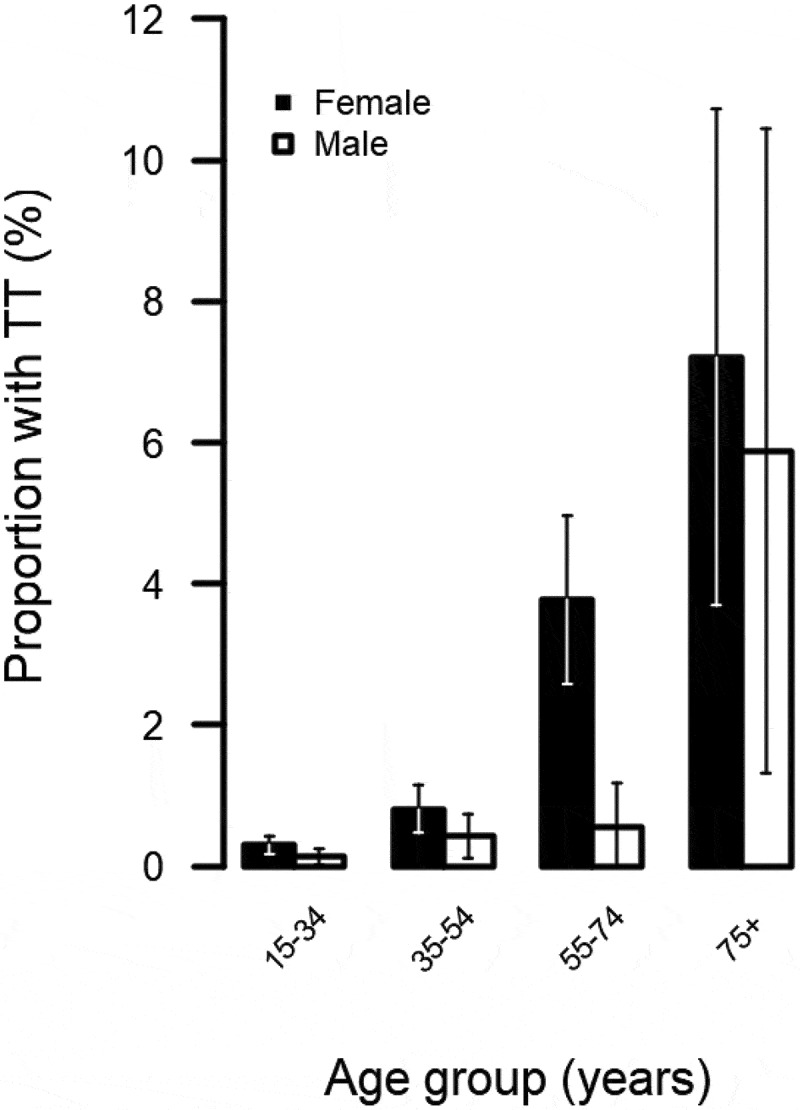
Proportion of refugee settlement residents in Uganda with trachomatous trichiasis (TT; lid and management status not specified) in different age groups, April 2018–February 2020. Whiskers represent 95% confidence intervals around age-specific proportion estimates.

The number of adults with TT and the adjusted prevalence of TT unknown to the health system is shown in Table 3. Disease prevalence in each EU is visualised in Figure 1. In the six EUs surveyed in 2018, TT was above the WHO elimination threshold of 0.2%; however, the eyelid(s) of origin of in-turned eyelash(es) was not recorded. In these EUs, 102 cases of TT (eyelid not specified) were identified, of which 88 had concurrent TT and TS. Of the 102 people with TT, 91 had a least one eye with TT for which management had not previously been offered, so were categorised as unknown to the health system. In the five EUs surveyed in 2020, the eyelid of origin of the misdirected lashes was recorded. Teams identified 13 cases of upper-eyelid TT, of which 12 were unknown to the health system. There were three participants in the five EUs surveyed in 2020 with lower-eyelid trichiasis, one of whom had no upper-eyelid involvement. TT (eyelid and management status not specified) was more common in older people (compared to the reference age group of 15–34-year-olds, adjusted OR [aOR] for 35 − 54-year-olds: 2.9, 95% CI: 1.7 − 4.9; for 55 − 74-year-olds: 11.7, 95% CI: 7.1 − 19.4; for ≥75-year-olds: 30.7, 95% CI: 16.7 − 56.4; Figure 2; Supplementary Table 2) and in females compared to males (aOR: 2.5, 95% CI: 1.6–4.0, *p* < .001; Supplementary Table 2).

**Table 3.** Participants aged ≥15 years with trichiasis and adjusted prevalence of trachomatous trichiasis (TT) unknown to the health system in refugee settlements in Uganda, April 2018–February 2020.

Region	District hosting refugee settlements	Examined	Trichiasis (eyelid not specified) and TS	Trichiasis (eyelid not specified)	Trichiasis (eyelid not specified) unknown to the health system	Upper-eyelid trichiasis	Upper-eyelid trichiasis unknown to the health system	Adjusted prevalence of trichiasis (eyelid not specified) unknown to the health system (%, 95% CI)	Adjusted prevalence of upper-eyelid trichiasis unknown to the health system (%, 95% CI)
Northern	Arua	1,696	9	9	8	-	-	0.35 (0.03–0.89)	-
Northern	Yumbe	1,749	5	6	6	-	-	0.41 (0.05–1.1)	-
Northern	Moyo	1,651	10	10	10	-	-	0.42 (0.18–0.68)	-
Northern	Adjumani	1,462	22	27	24	-	-	0.53 (0.22–0.97)	-
Northern	Lamwo	1,500	13	16	13	-	-	0.51 (0.23–0.91)	-
Western	Kiryandongo	1,588	29	34	30	-	-	1.4 (0.66–2.2)	-
Northern	Koboko	970	-	-	-	3	3	-	0.13 (0.00–0.28)
Western	Isingiro	1,750	-	-	-	2	2	-	0.05 (0.00–0.12)
Western	Kamwengye	1,659	-	-	-	1	1	-	0.03 (0.00–0.09)
Western	Kikuube	1,503	-	-	-	5	4	-	0.10 (0.01–0.19)
Western	Kyegegwa	1,609	-	-	-	2	2	-	0.05 (0.00–0.12)
*Total*		*17,137*							

-: not reported.

CI: confidence interval; TS: trachomatous scarring.

### WASH infrastructure coverage

The household-level water and waste management in these refugee settlements varied between EUs. The range in EU-level proportion of households with an improved drinking water source was 67–100%. Notably, in five EUs, 100% of households had access to an improved water source, although it took approximately half the households in those EUs >30 minutes (including waiting time) to get drinking water (Table 4). The median proportion of households per EU with an improved latrine was 30% (range: 17–88%) (Table 4). Handwashing facility access was more limited across the study areas (Table 4).

**Table 4.** Water, sanitation and hygiene access for households in refugee settlements during baseline trachoma surveys in Uganda, April 2018− February 2020.

Region	District hosting refugee settlements	Primary Sampling Units	Households	Number of households with …							
				Improved drinking water source (%)	Improved washing water source (%)	Drinking water source within 30-minute round trip (%)	Washing water source within 30-minute round trip (%)	Improved latrine (%)	Latrine with handwash station (%)	Handwash station on the premises (%)	Improved drinking water source within 30-minute round trip (%)
Northern	Arua	24	843	808 (96)	808 (96)	474 (56)	427 (51)	301 (36)	381 (45)	-	441 (52)
Northern	Yumbe	24	847	846 (100)	846 (100)	451 (53)	377 (45)	257 (30)	378 (45)	-	451 (53)
Northern	Moyo	24	844	844 (100)	844 (100)	370 (44)	338 (40)	147 (17)	208 (25)	-	370 (44)
Northern	Adjumani	24	841	840 (100)	841 (100)	422 (50)	375 (45)	235 (28)	182 (22)	-	421 (50)
Northern	Lamwo	24	843	843 (100)	843 (100)	416 (49)	357 (42)	212 (25)	231 (27)	-	416 (49)
Western	Kiryandongo	24	848	848 (100)	848 (100)	274 (32)	255 (30)	255 (30)	-	75 (9)	274 (32)
Northern	Koboko	18	549	534 (97)	533 (97)	262 (48)	255 (46)	163 (30)	-	35 (6)	247 (45)
Western	Isingiro	30	911	682 (75)	646 (71)	248 (27)	255 (28)	798 (88)	-	61 (7)	232 (25)
Western	Kamwengye	30	911	784 (86)	771 (85)	236 (26)	232 (25)	733 (80)	-	23 (3)	215 (24)
Western	Kikuube	30	919	616 (67)	614 (67)	252 (27)	243 (26)	356 (39)	-	52 (6)	159 (17)
Western	Kyegegwa	30	918	746 (81)	723 (79)	271 (30)	285 (31)	318 (35)	-	48 (5)	237 (26)

## Discussion

The potential risk of re-introduction of ocular *C. trachomatis* infection into treated communities following cross-border migration from the neighbouring trachoma-endemic countries of DRC and South Sudan warranted surveys of trachoma in refugee populations displaced into Uganda. Here, the prevalence of TF in 1–9-year-olds was <5% in all 11 EUs studied, suggesting antibiotic MDA is not warranted in these populations for trachoma elimination purposes. If the prevalence of trachoma was high in the surrounding host district communities, there may be a risk of seeding of new infections into the refugee population. According to the Global Trachoma Atlas (www.trachomaatlas.org), the prevalence of TF in children in the resident Ugandan population was <5% at most recent survey in host districts in the Northern Region. Other host districts (Isingiro, Kamwengye, Kikube, Koboko and Kyegegwa) have not recently been surveyed because they are not suspected to be endemic for trachoma. The risk of seeding of new infections from surrounding communities into refugee settlements is therefore expected to be low.

Prevalence of TT unknown to the health system was ≥0.2% in six (five Northern, one Western) refugee settlement EUs; therefore, active identification and management of TT is needed in these areas. Assuming 40% of the population are aged ≥15 years, we might expect there to be ~2,100 trichiasis cases across all these six EUs (calculated using the approach suggested by Solomon *et al*. and in acknowledgement of the inherent inaccuracy in such estimates.^30^) Currently, these services are delivered through village health teams in each block who are trained as TT case finders. When village health teams encounter an individual with TT, they are referred to the Ophthalmic Clinical Officer at the local health facility, who confirms the diagnosis and registers the individual for management at annual outreach clinics. There are also annual case finding campaigns. The best method to deliver these services sustainably may differ from the method used to deliver surgical services to the permanently resident communities of host districts and will require close collaboration between the Uganda Ministry of Health and administrative bodies supporting refugees. The requirements for ongoing surveillance of these settlements are also likely to differ from those applying to permanently resident populations, due to higher rates of in- and out-migration in refugee settlements. The reasons for the higher TT prevalence in the six EUs with a TT prevalence >0.2% are not clear. Because TT takes many years to develop, the data collected in these cross-sectional surveys are unlikely to be able to capture the complex accumulated risk factors from the lifetime of an individual with TT, especially because we collected no additional data on participants living situation prior to arrival at the settlement. We were also unable to generate hypotheses on the reasons for the higher TT prevalence through review of routinely available UNHCR data from the settlements (data2.unhcr.org/en/country/uga).

Given the high prevalence of trachoma in South Sudan^12,31^ in particular, it is surprising that the prevalence of TF among the refugee population was so low. This low prevalence has been noted in other settlements serving refugees from South Sudan, for example in Sudan and in Ethiopia.^32,33^ A number of factors may contribute. First, interactions between children and therefore transmission dynamics may differ in refugee settlements where there is more resident turnover and less familiarity between neighbours. Second, the poorest and most vulnerable among society may be simultaneously more likely to have trachoma and less likely to leave their original country of residence,^34^ thereby reducing the influx of affected individuals into refugee camps. Finally, it is notable that the majority of households had good access to improved water sources in this study, a finding which matches closely with UNHCR monitoring data from some of these settlements (wash.unhcr.org/wash-dashboard-for-refugee-settings/). There may also be other infrastructure components not recorded in these surveys which help people maintain facial cleanliness and thereby reduce risk of acquiring trachoma. (It should be noted that in Sudan, the odds of having TF were 2.6-times higher in children living in internally-displaced person camps than in children living elsewhere, so the low risk noted in this paragraph is not universal.)^29^

Despite the relatively low prevalence, the age distribution of trachoma in these populations was typical of other more heavily trachoma-endemic populations – TF was most common in pre-school age children and TT was most common in the oldest adults.^29,35–37^ TT also disproportionately affected women^38^ in these surveys. In addition, there was some marginal evidence that TF was more common in children living > 30 minutes away from a drinking water source (aOR 1.27; 95% CI: 0.98–1.64, *p* = .051) and in children using surface water for drinking water (aOR: 1.93; 95% CI: 1.12–3.32, *p* = .051). There is particular room for improvement in the proximity of water sources to household, the provision of improved latrines and the provision of handwash facilities in these settlements which should be rectified, not just from the point of view of preventing trachoma.^39^

We find that refugee settlements in Uganda are not in need of antibiotic MDA, facial cleanliness promotion or environmental improvement for the purposes of trachoma’s elimination as a public health problem. However, due to the mobile nature of displaced populations, this situation could change if the population of these settlements’ changes. Therefore, improvements to WASH facilities which may overlap with F and E activities may still be valuable. The 2021–2030 NTD roadmap recommends targeting 100% access to basic WASH facilities as part of an integrated and sustainable NTD control strategy,^5^ an approach which has been embraced in Uganda’s most recent sustainability plan for NTD control, released in February 2021.^40^ Furthermore, active case identification and management of TT is needed in six of the EUs. It is likely that refugee settlements may need more regular surveillance than permanently inhabited neighbouring areas. Close collaboration between health authorities and partners could enable trachoma screening to be integrated with routine health provision in refugee settlements. Innovative solutions such as these are required to ensure that the health of refugees and the surrounding communities is maintained.

## Conclusions

Implementation of the antibiotic, facial cleanliness and environmental improvement components of the SAFE strategy is not needed for the purposes of trachoma’s elimination as a public health problem in these refugee settlements; however, intervention with TT surgery is needed in six EUs. Since instability continues to drive displacement of people from South Sudan and DRC into Uganda, there is likely to be a high rate of new arrivals to the settlements over the coming years. These populations may therefore have trachoma surveillance needs that are distinct from the surrounding non-refugee communities.
